# Pathogenesis and Pharmacotherapy of Acute Respiratory Distress Syndrome Induced by Pandemic Viral Infections: A Narrative Review

**DOI:** 10.7759/cureus.101316

**Published:** 2026-01-11

**Authors:** Luiza Gabunia, Shorena Khetsuriani, Natia Gamkrelidze, Nino Gvajaia, Levan Ratiani, Gvantsa Janigashvili

**Affiliations:** 1 Department of Clinical Pharmacology, Tbilisi State Medical University, Tbilisi, GEO; 2 Department of Microbiology, Tbilisi State Medical University, Tbilisi, GEO; 3 Department of Pathophysiology, Tbilisi State Medical University, Tbilisi, GEO; 4 Department of Medicine, Tbilisi State Medical University, Tbilisi, GEO; 5 Department of Intensive Care Unit, The First University Clinic of Tbilisi State Medical University, Tbilisi, GEO

**Keywords:** acute respiratory distress syndrome, pandemic viruses, pathogenesis of ards, pharmacotherapy of ards, viral pathogenesis

## Abstract

Acute respiratory distress syndrome (ARDS) is a severe, life-threatening condition characterized by acute hypoxemic respiratory failure, bilateral pulmonary infiltrates, and non-cardiogenic pulmonary edema caused by increased alveolar-capillary permeability. ARDS is highly heterogeneous, with diverse etiologies and clinical presentations that complicate diagnosis and management. Viral infections, including influenza A (H1N1 and H5N1) and coronaviruses such as SARS-CoV-2, are major contributors to ARDS and can trigger severe lung injury, hyperinflammation, and dysregulated immune responses. Ongoing viral evolution and periodic emergence of novel strains continue to pose a substantial threat to global public health. This narrative review analyzes pandemic-associated viral causes of ARDS, summarizes key mechanisms of pathogenesis, and evaluates current and emerging pharmacotherapeutic approaches. A comprehensive literature search was conducted using PubMed, supplemented by additional sources where appropriate. The review highlights that the increasing prominence of viral pneumonia as a cause of ARDS requires both established supportive care and tailored therapeutic strategies that target the underlying mechanisms of lung injury. Despite progress, virus-associated ARDS remains a major clinical challenge with high morbidity and mortality and may require management approaches distinct from those used for other ARDS etiologies.

## Introduction and background

Acute respiratory distress syndrome (ARDS) is a severe form of acute hypoxemic respiratory failure that develops after direct pulmonary injury (e.g., pneumonia and aspiration) or indirect systemic insults (e.g., sepsis and trauma). It is characterized by diffuse inflammatory lung injury with increased permeability of the alveolar-capillary barrier, resulting in noncardiogenic pulmonary edema, reduced lung compliance, and impaired gas exchange. Clinically, these changes manifest as profound hypoxemia driven by intrapulmonary shunt and ventilation-perfusion mismatch [1,2].

Because no single test confirms ARDS, diagnosis relies on clinical and radiographic criteria. The Berlin Definition (2012) remains the most widely used framework and requires onset within seven days of a known insult, bilateral opacities on chest imaging not fully explained by effusions, collapse, or nodules, and respiratory failure not primarily attributable to cardiac failure or fluid overload. Severity is stratified using the arterial partial pressure of oxygen to fraction of inspired oxygen ratio (PaO₂/FiO₂) in patients receiving at least 5 cm H₂O of positive end-expiratory pressure, with lower ratios indicating more severe oxygenation impairment [3].

ARDS continues to impose a major global burden in intensive care settings, with substantial mortality and long-term morbidity among survivors. Outcomes are influenced by patient factors such as age and comorbidities, as well as by the underlying cause and severity of lung injury. Despite advances in lung-protective ventilation and supportive care, ARDS remains a leading cause of death from critical illness and a contributor to prolonged functional impairment after discharge [1,4-7].

Importantly, ARDS is increasingly recognized as a heterogeneous syndrome rather than a single disease entity. Distinct clinical and biological subphenotypes have been described that differ in inflammatory profiles, radiographic patterns, and responses to therapy. This heterogeneity complicates both bedside management and drug development because interventions may benefit some subgroups while offering limited efficacy or causing harm in others [3-6].

This narrative review summarizes current concepts in ARDS pathogenesis and highlights how emerging and pandemic respiratory viral infections have shaped contemporary understanding of the syndrome. It also reviews current and evolving pharmacotherapeutic approaches, with particular attention to therapies studied in viral-associated ARDS and potential phenotype-directed strategies.

## Review

Review methodology

A narrative literature review was conducted to summarize current evidence on the pathogenesis and pharmacotherapy of ARDS caused by pandemic viral infections. We searched PubMed/MEDLINE and Google Scholar for English-language, peer-reviewed publications from January 2000 through December 2025 using combinations of keywords and MeSH terms including “acute respiratory distress syndrome”, “ARDS”, “viral ARDS”, “influenza”, “H1N1”, “H5N1”, “SARS-CoV-2”, “COVID-19”, “pathogenesis”, “endothelial injury”, “biomarkers”, “immunomodulators”, “corticosteroids”, “interleukin-6”, “Janus kinase inhibitors”, and “antiviral therapy”. Priority was given to high-quality evidence (clinical practice guidelines, randomized trials, systematic reviews/meta-analyses, and large observational cohorts), complemented by key mechanistic and translational studies relevant to epithelial-endothelial injury, immune dysregulation, and immunothrombosis. Additional relevant articles were identified by screening the reference lists of major reviews and guideline documents. Studies were selected for inclusion based on relevance to the review aims (pandemic viral etiologies of ARDS, mechanisms of lung injury, and therapeutic strategies) and the strength and recency of the evidence.

Because this work was designed as a narrative review intended to integrate mechanistic and clinical evidence, it was not conducted as a Preferred Reporting Items for Systematic reviews and Meta-Analyses (PRISMA)-guided systematic review and therefore did not apply formal study-level inclusion/exclusion criteria or a quantitative synthesis.

Pandemic respiratory viruses: from viral evolution to systemic disease

Respiratory virus evolution is shaped by frequent genetic changes, including recombination and reassortment, which have repeatedly driven epidemics and pandemics and contributed to substantial global mortality. Among contemporary viral threats, influenza A viruses and SARS-CoV-2 remain major public health challenges in the 21st century [8-10]. Both viruses continue to impose a large global disease burden, with influenza causing extensive annual infections and SARS-CoV-2 sustaining widespread transmission since 2019 [11-13]. According to the Centers for Disease Control and Prevention, hospitalization and mortality rates from influenza and COVID-19 are higher in adults aged 50 years and older [14].

Among the diverse etiologies of ARDS, respiratory viruses play a prominent role because some pathogens demonstrate a marked propensity to cause severe pneumonia that progresses to ARDS. Several high-impact viral events, such as MERS-CoV, SARS-CoV in 2002-2003, avian influenza A/H5N1, and influenza A/H1N1 in 2009, have been central drivers of acute respiratory failure and ARDS in prior outbreaks [6,15-17].

Historically, influenza A viruses have been responsible for more documented global pandemics than any other infectious agent [18]. The highly pathogenic avian influenza A/H5N1 virus has repeatedly crossed species barriers to infect humans and has caused outbreaks in numerous countries [19]. Since its emergence in 1997, reported human infections have been associated with mortality exceeding 50% in documented cases [20]. Understanding the recent expansion of the H5N1 host range and evaluating plausible animal-to-human transmission pathways remain important for assessing pandemic potential, particularly as newly emerging reassortant H5N1 strains appear more capable of infecting a broader range of mammalian hosts, including humans [21]. In parallel, human H5N1 cases have increased alongside growing exposure to infected animals, sustaining concern about future outbreaks and the potential for pandemic spread [22]. Since H5N1 was recognized as capable of infecting humans, multiple reports have described ARDS resulting from infection [23].

Influenza A viruses have repeatedly caused pandemics through antigenic drift and reassortment, enabling adaptation to human hosts and periodic emergence of novel strains. Historically, influenza A viruses have been responsible for more documented global pandemics than any other respiratory pathogen, reflecting their genetic plasticity and broad host range. [9]. Pandemic strains have typically arisen through reassortment between avian, swine, and human influenza viruses, resulting in efficient human transmission and, in some cases, severe lower respiratory tract disease and ARDS. Contemporary concern remains focused on highly pathogenic avian influenza strains such as H5N1, which continue to circulate globally in avian and mammalian reservoirs and have demonstrated the capacity to cause severe viral pneumonia and ARDS in humans [18].

SARS-CoV-2 was first identified in Wuhan, China, in December 2019 and rapidly spread worldwide. WHO declared COVID-19 a global pandemic on March 11, 2020 [24]. Since then, multiple variants have emerged with altered transmissibility and pathogenicity [25-27]. WHO classifies circulating strains as variants under monitoring, variants of interest, or variants of concern [24]. Viral entry is mediated by binding of the spike protein to the angiotensin-converting enzyme 2 (ACE2) receptor, which is highly expressed in pulmonary epithelium and vascular endothelium, contributing to severe lung injury and multiorgan involvement [26,28].

Influenza infection can cause systemic complications beyond pneumonia, including ARDS and encephalitis [29]. Clinical evidence supports multi-organ involvement affecting the heart, brain, kidneys, liver, muscles, eyes, and hematologic system. Viral myocarditis, viral encephalitis, and liver injury are among the most commonly reported complications [10]. Influenza also increases susceptibility to severe bacterial complications such as sepsis and secondary bacterial pneumonia, most commonly caused by Staphylococcus aureus or Streptococcus pneumoniae, which can further precipitate ARDS [8,30].

Although COVID-19 primarily affects the respiratory system, severe disease is often characterized by systemic inflammation and hyperinflammatory syndromes, including cytokine storm, which contribute to myocardial injury and other complications [31]. Cardiac involvement, including ischemia or infarction and myocarditis, has been reported, and elevated troponin levels have been associated with a higher risk of malignant arrhythmias and an increased need for mechanical ventilation in hospitalized cohorts. Acute kidney injury is a common extrapulmonary complication and is associated with increased mortality. Hematologic abnormalities such as lymphopenia, thrombocytopenia, leukopenia, and elevated inflammatory markers (e.g., erythrocyte sedimentation rate, C-reactive protein, and lactate dehydrogenase) are frequently observed. COVID-19 is also associated with a hypercoagulable state, reflected by elevated D-dimer and fibrinogen levels, prolonged coagulation times, and an increased risk of venous thromboembolism. Gastrointestinal manifestations (e.g., diarrhea, nausea, vomiting, anorexia, and abdominal pain) are common, and rare thrombotic events such as mesenteric ischemia and portal vein thrombosis have been described. Liver enzyme elevations are frequently reported, and neurological and cutaneous complications have also been documented [26].

Pathophysiology of virus-induced ARDS: cellular and molecular mechanisms

Although viral pathogens differ in structure, tropism, and immune evasion strategies, many downstream mechanisms of lung injury converge on shared ARDS pathways. Direct viral cytopathic effects, epithelial and endothelial barrier disruption, dysregulated innate and adaptive immune responses, and activation of immunothrombotic cascades represent common final pathways leading to alveolar flooding, hypoxemia, and respiratory failure. To avoid redundancy, the following section integrates virus-specific features with these shared ARDS mechanisms, highlighting points of overlap while emphasizing distinctions that may influence therapeutic response.

Respiratory viruses continue to generate recurrent endemic and pandemic threats, with clinical outcomes ranging from mild upper respiratory illness to life-threatening ARDS [32]. ARDS remains a critical illness associated with substantial acute complications and long-term disability. In the acute phase, patients may require prolonged mechanical ventilation for refractory respiratory failure, sometimes necessitating tracheostomy. Extended immobilization and exposure to the intensive care environment can contribute to delirium, critical illness myopathy or polyneuropathy, and secondary nosocomial infections. Among survivors, chronic sequelae can markedly reduce quality of life and include irreversible structural changes such as pulmonary fibrosis and tracheal stenosis, as well as impaired lung function, persistent muscle weakness, and ambulatory dysfunction. Neurocognitive and psychological effects, including cognitive impairment, memory deficits, post-traumatic stress disorder, depression, and anxiety, may persist for years after hospitalization. Despite improvements in survival over recent decades, mortality remains high, with estimates near 40%, and the dominant causes of death have remained largely unchanged since the 1980s, most notably sepsis or septic shock and subsequent multi-organ failure [33].

The pathogenesis of ARDS is multifactorial and reflects dysregulation across interconnected pulmonary and systemic pathways involving tissue injury, inflammation, and coagulation. Injury to multiple cell types, including alveolar epithelial cells, pulmonary endothelial cells, macrophages, and fibroblasts, contributes to barrier dysfunction, pulmonary edema, and respiratory failure [6]. A central physiologic hallmark of ARDS is the formation of protein-rich pulmonary edema, which compromises oxygenation and impairs carbon dioxide elimination, leading to profound hypoxemia and ventilatory failure [7]. At the molecular level, ARDS is closely linked to an aggressive inflammatory response mediated by cytokines such as tumor necrosis factor-alpha (TNF-α), IL-1, and IL-6. These mediators amplify inflammatory cascades, recruit immune cells, and exacerbate tissue injury. Importantly, this inflammatory response is not confined to the lungs; systemic cytokine activity, often described as a “cytokine storm,” correlates with worse clinical outcomes and can be fatal [7,16].

Clinically and pathologically, ARDS progresses through overlapping phases commonly described as exudative, proliferative, and fibrotic. The exudative phase typically occurs within the first week of illness and is characterized by widespread inflammation and injury to endothelial and epithelial barriers, allowing leakage of protein-rich fluid into the alveoli and resulting in pulmonary edema and severe hypoxemia [34]. Around days 7-10, the proliferative phase begins as type II pneumocytes proliferate and attempt to restore epithelial integrity while alveolar edema gradually resolves. However, resolution may be incomplete when epithelial injury delays alveolar fluid clearance and reduces functional surfactant. In prolonged or severe disease, a fibrotic phase may ensue, characterized by irreversible scarring and architectural remodeling that drives chronic impairment and reduced long-term quality of life among ARDS survivors [1,7].

Arterial blood gas analysis is used to assess the severity of hypoxemia in ARDS, typically demonstrating reduced partial pressure of arterial oxygen. Early in the disease course, hyperventilation may produce respiratory alkalosis, whereas later progression and ventilatory failure can lead to respiratory acidosis as carbon dioxide retention develops. In parallel, research has explored biomarkers for diagnosis and prognosis, including surfactant proteins, cytokines, and markers of endothelial injury. Elevated inflammatory cytokines such as IL-6 and IL-8 are frequently observed and may support early recognition or future risk stratification [35]. Although several inflammatory and endothelial biomarkers have demonstrated prognostic and phenotypic associations in ARDS, most are not yet recommended for routine clinical decision-making and remain primarily investigational. From a diagnostic standpoint, distinguishing ARDS from other causes of respiratory distress is essential, as conditions such as cardiogenic pulmonary edema, pneumonia, and diffuse alveolar hemorrhage may present with similar clinical features but require different management strategies. Clinical history, echocardiography, and hemodynamic assessment are particularly important in differentiating noncardiogenic pulmonary edema, which is typical of ARDS, from heart failure-related pulmonary edema [1].

In influenza-associated lung injury, severe respiratory failure arises from a combination of direct viral injury to the respiratory epithelium and immune-mediated inflammation. Viral replication in respiratory tissues is facilitated when hemagglutinin is effectively cleaved, enabling production of infectious virions [36]. The magnitude of the inflammatory response varies by strain; H5N1 can provoke stronger inflammatory activation than H1N1pdm09 and H7N7 in blood macrophages [8]. Mechanistically, H5N1 has been shown to disrupt alveolar epithelial integrity through autophagy-mediated cell death and degradation of junction proteins, increasing epithelial permeability, promoting edema and inflammatory infiltration, and compromising the alveolar-capillary barrier. This barrier disruption may increase susceptibility to secondary infections and facilitate viral dissemination beyond the respiratory tract [20,37]. The 2009 H1N1 pandemic highlighted that influenza can precipitate unusually severe, therapy-refractory ARDS, particularly among individuals lacking preexisting immunity [38]. Influenza A has been described as a leading viral cause of ARDS in adults, with risk factors including age 36-55 years, pregnancy, and obesity, and with disease driven by both direct viral cytopathic effects and immunopathology [8]. Influenza targets alveolar epithelial cells, including type I and type II pneumocytes, and high viral loads combined with robust host responses can lead to airway obstruction, alveolar destruction, epithelial cell death, and extracellular matrix breakdown, culminating in impaired gas exchange and ARDS [8,39].

The COVID-19 pandemic emphasized the capacity of SARS-CoV-2 to trigger viral ARDS at scale. SARS-CoV-2-associated ARDS has been particularly prominent among vulnerable patients and has been described as a leading global contributor to ARDS during the pandemic era [32]. Published data indicate that a substantial proportion of patients with COVID-19 pneumonia progressed to ARDS, with many requiring intensive care [40-41]. During the first pandemic year in the United States, ARDS-related mortality increased markedly. Systematic review evidence has also supported a high pooled ARDS prevalence among patients with COVID-19 [27]. Although vaccination and evolving viral variants altered later patterns of disease severity, SARS-CoV-2 remained a major cause of critical respiratory illness during multiple waves [42].

Mechanistically, COVID-19 lung injury is initiated by direct viral cytopathic effects and is then compounded by dysregulated host responses that include inflammatory and thrombotic processes within pulmonary and extrapulmonary compartments. Viral entry occurs through binding to ACE2 receptors expressed on the alveolar epithelium and vascular endothelium. This interaction can produce cellular injury, interstitial edema, and alveolar flooding that resemble the alveolar fluid accumulation typical of classical ARDS [2]. Because ACE2 normally degrades pro-inflammatory angiotensin II, viral downregulation or inhibition of ACE2 may increase angiotensin II levels and contribute to systemic inflammation and cytokine cascades that drive progression to ARDS and multi-organ dysfunction [2,43]. During early infection, viral replication predominates in tissue injury, whereas later stages may be characterized by recruitment of immune cells and release of pro-inflammatory mediators such as TNF-α, granulocyte-macrophage colony-stimulating factor, IL-1, IL-6, IL-1β, IL-8, IL-12, and interferon-gamma. In severe cases, immune dysregulation can produce a cytokine storm with both localized and systemic tissue injury [26]. Cytokine storm refers to a sudden, excessive surge of cytokines reflecting immune overactivation that can progress to multi-organ failure and death without timely intervention [28].

Outcomes are especially poor in individuals with cardiovascular and metabolic comorbidities such as hypertension and diabetes, suggesting that baseline immune dysregulation or heightened procoagulant states may increase vulnerability to complications. Post-infection cardiac sequelae, including heart failure and myocarditis, have been reported even in nonhospitalized, vaccinated individuals, indicating that inflammatory and vascular effects can remain clinically relevant beyond acute hospitalization [44]. Proposed mechanisms include destabilization of vascular plaques during systemic inflammation, increased cardiac demand during acute infection, and potential direct myocardial injury mediated through ACE2 expression in cardiac tissue [43].

Although SARS-CoV-2 lung injury shares key features with other forms of ARDS, early clinical presentation has sometimes differed, including profound hypoxemia with comparatively limited perceived dyspnea despite modest initial imaging findings. Autopsy findings often demonstrate diffuse alveolar damage across exudative, proliferative, and fibrotic phases, along with alveolar and interstitial edema, hemorrhage, endothelial injury, capillary congestion, microthrombosis, and vascular dilation. A distinguishing feature described in COVID-19 and earlier SARS is more prominent vasculopathy compared with H1N1 influenza and classic ARDS, including widespread macrothrombosis and microthrombosis, endothelial injury, vascular dilation, and aberrant angiogenesis. However, early-stage biopsies do not always show the same extent of vascular pathology observed later at autopsy. Bronchoalveolar lavage studies have suggested an airspace cell profile dominated by monocytes and lymphocytes, consistent with viral pneumonia patterns rather than the neutrophil predominance often observed in classic ARDS. A clearer understanding of these physiologic differences remains important for improving clinical management and supportive care strategies [2].

Evidence and future directions of ARDS pharmacotherapy

Supportive and Ventilator-Based Management

For decades, ARDS management has centered on supportive, nonpharmacologic approaches intended to optimize oxygenation and limit ventilator-induced lung injury. Core strategies include lung-protective mechanical ventilation, prone positioning, and, in selected cases, extracorporeal support. Contemporary ARDS care remains multifaceted, combining ventilatory management, pharmacologic interventions, and life-support strategies such as extracorporeal membrane oxygenation when indicated [1].

Immunomodulatory Therapies

Clinical guidance includes recommendations across pharmacologic and ventilatory domains. Among pharmacologic therapies, corticosteroids are suggested for patients with ARDS as a conditional recommendation supported by moderate-certainty evidence [45]. Randomized trial data suggest that early dexamethasone may shorten the duration of mechanical ventilation and reduce mortality among patients with established moderate-to-severe ARDS [46]. At the same time, the search for effective pharmacotherapies in virus-induced lung injury remains challenging because key mechanisms of epithelial-endothelial injury, inflammatory dysregulation, thrombosis, and fibrotic remodeling are incompletely characterized, and continued viral evolution can limit the durability and generalizability of any single strategy across outbreaks and patient populations [47].

Endothelial-Targeted and Adjunctive Therapies

In addition to supportive care, therapies targeting immune dysregulation have been explored because cytokine-driven injury and sustained inflammatory activation contribute to the pathogenesis of viral ARDS. Investigational and repurposed immunomodulators include cytokine-directed interventions such as TNF-α inhibition (e.g., GSK 1995057), IL-1 blockade with anakinra, IL-6 receptor antagonists such as tocilizumab and sarilumab, and broader intracellular pathway modulation with Janus kinase inhibitors (e.g., baricitinib). Neutrophil elastase inhibition with sivelestat has also been examined as a strategy to reduce neutrophil-mediated tissue injury and preserve the alveolar-capillary barrier. In parallel, vascular and endothelial protection has drawn attention because permeability injury, pulmonary edema, and hypercoagulability are prominent features of severe viral ARDS. Candidate approaches include Abl kinase inhibition (imatinib), statins, anticoagulants, and investigational endothelial barrier stabilizers, with the goal of reducing vascular leak and thrombotic complications. Adjunctive therapies have also been considered in selected settings. Macrolides may exert immunomodulatory effects in pneumonia and ARDS, potentially improving early clinical response, and short-term neuromuscular blockade with agents such as cisatracurium can be used in carefully selected patients to improve ventilator synchrony and may reduce ventilator-induced lung injury, although careful monitoring and frequent reassessment remain essential. Corticosteroids continue to be discussed as a key anti-inflammatory intervention, with outcomes dependent on timing, dose, and underlying etiology [48].

Because lung inflammation and progression to pulmonary fibrosis contribute to long-term morbidity, therapeutic strategies aimed at mitigating inflammatory injury and limiting fibrotic progression remain of interest as complements to antiviral therapy. Interventions intended to reduce lung injury may be most effective when acting locally at the site of pathology; therefore, inhaled or intratracheal delivery of anti-inflammatory agents, antioxidants, hormones, or other bioactive compounds has been proposed to enhance local efficacy while reducing systemic exposure and minimizing off-target adverse effects in other organs [49]. Although the mechanisms underlying cytokine storm are not fully clarified, immunomodulatory approaches remain a plausible strategy for attenuating hyperinflammation in severe acute viral infections [16].

Antiviral Therapy

Antiviral treatment remains a central component of management in virus-associated ARDS, particularly in influenza-related disease. Importantly, influenza-associated ARDS illustrates that immunomodulation can carry etiology-specific risks. Multiple studies have reported that early corticosteroid therapy in influenza-associated ARDS is associated with increased hospital mortality and higher odds of secondary bacteremia, supporting recommendations against routine corticosteroid use in this setting when benefit has not been demonstrated and infectious risks may be increased [17,50]. Meta-analyses evaluating corticosteroids in influenza-associated ARDS and severe pneumonia have similarly reported increased mortality and higher rates of nosocomial infection [51]. Registry-based analyses from the 2009 H1N1 pandemic also associated early corticosteroid therapy with increased hospital mortality, with particularly unfavorable outcomes when administered early [52,53]. Importantly, early initiation of antiviral therapy, particularly neuraminidase inhibitors such as oseltamivir, remains the cornerstone of management for influenza-associated ARDS, while adjunctive immunomodulatory therapies should be considered cautiously and on a case-by-case basis. Some small observational reports described favorable outcomes with combined oseltamivir and prolonged low-to-moderate corticosteroid regimens; however, conclusions remain limited by small sample size and the absence of control groups [53]. For H5N1-associated ARDS, management remains largely supportive, and current guidance emphasizes neuraminidase inhibition, such as oseltamivir, with consideration of zanamivir in oseltamivir-resistant strains, for which reported outcomes have been encouraging in severely ill patients [17].

COVID-19-Specific Considerations and Emerging Evidence

In COVID-19, further advances in therapy require continued clarification of SARS-CoV-2-associated ARDS pathogenesis, including the interplay of viral replication, immune dysregulation, endothelial injury, thrombosis, and barrier disruption. Established ARDS pharmacotherapies such as corticosteroids and statins have been widely used; however, their efficacy and safety in COVID-19 have been debated with respect to dose, timing, and disease stage, underscoring the need for additional clinical research to refine treatment strategies [48]. Evidence syntheses have shown variability across study designs: randomized trials have demonstrated benefits in selected contexts, whereas observational studies have suggested potential risks depending on steroid type and timing. Meta-analyses have reported that corticosteroids probably reduce mortality in ARDS of multiple etiologies, including COVID-19, and have been associated with improvements in ventilator-free days in some trials [54,55]. A 2021 meta-analysis further suggested that steroid type, dose, and timing influence outcomes and may reduce mortality across ARDS etiologies [56]. At the same time, systematic reviews have noted uncertainty regarding long-term effects, even when short-term mortality reductions appear limited in some analyses [57].

The COVID-19 pandemic also renewed interest in host-targeted therapies, including repurposed immunomodulators and endothelial-stabilizing agents, and results from COVID-19 ARDS trials may inform treatment paradigms beyond COVID-19 itself [57]. For example, intravenous imatinib did not improve overall outcomes or broadly reduce pulmonary edema among mechanically ventilated patients with COVID-19; however, potential benefit in a subgroup suggests that future trials may require improved phenotype-based targeting [58]. Pre-pandemic ARDS research suggested a potential mortality benefit from higher-dose dexamethasone in some contexts, contributing to multiple trials evaluating high-dose corticosteroid regimens in COVID-19 [59,60]. High-dose dexamethasone has been associated with shorter durations of mechanical ventilation and improved survival in some COVID-19 ARDS cohorts, whereas larger meta-analyses have reported increased ventilator-free days with higher-dose corticosteroids but minimal overall mortality impact in certain syntheses [54,61,62].

As COVID-19 therapeutics evolved, management increasingly relied on antivirals and immunomodulators. Antivirals such as remdesivir and ritonavir appear to provide the greatest benefit early in the disease course, whereas immunomodulators such as dexamethasone and inhibitors of IL-6 or Janus kinase pathways appear most beneficial in severe or critical illness. The effectiveness of anti-SARS-CoV-2 monoclonal antibodies has decreased as new variants have developed resistance, and convalescent plasma remains an area of uncertainty. Despite higher thromboembolic risk in COVID-19, antithrombotic approaches have shown limited effectiveness in some contexts, and multiple investigational therapies remain under study with the potential to reshape future practice [63,64].

Statins are widely prescribed for dyslipidemia and cardiovascular disease and have additional pleiotropic effects, including antioxidant, anti-inflammatory, and endothelial-protective properties that may be relevant to ARDS. Preclinical COVID-19 studies have suggested that statins could reduce pulmonary vascular complications by decreasing platelet aggregation and venous thrombosis and may also interfere with viral entry mechanisms. Retrospective analyses have reported potentially favorable associations with reduced COVID-19 mortality, complications, and hospitalization duration, raising the possibility that statins may be more beneficial in inflammatory phenotypes such as COVID-19-associated ARDS than in traditional ARDS populations, although causality remains uncertain [42].

Overall, persistent heterogeneity in virus-induced ARDS supports a move toward more personalized approaches that identify patient subgroups and inflammatory phenotypes to guide therapy selection. Ongoing research emphasizes phenotype-guided interventions, refined immune modulation, and novel pharmacologic strategies aimed at improving survival and reducing both acute and long-term complications in this complex patient population.

Future Directions and Precision-Based Management

Recent high-impact literature has further refined the conceptualization of ARDS, emphasizing its biological heterogeneity and the limitations of a single syndromic definition. Contemporary narrative and consensus reviews have proposed that ARDS should be understood as a spectrum of related biological subphenotypes rather than a uniform clinical entity, with important implications for prognosis, clinical trial design, and treatment selection. A recent Critical Care narrative review highlighted the need for future ARDS definitions that integrate clinical criteria with biological, radiographic, and molecular features, thereby enabling more tailored and mechanism-driven management strategies [65]. This evolving framework underscores why pharmacologic interventions may demonstrate variable efficacy across patient subgroups and supports a shift toward precision-based approaches in ARDS care.

In parallel, recent mechanistic studies have expanded understanding of the cellular and molecular features of COVID-19-associated ARDS. These investigations demonstrate that SARS-CoV-2-induced lung injury involves complex interactions between alveolar epithelial damage, endothelial dysfunction, dysregulated innate and adaptive immune responses, and immunothrombotic pathways [66]. Transcriptomic and single-cell analyses have identified distinct immune cell activation patterns, altered macrophage phenotypes, and persistent endothelial injury that differentiate COVID-19-associated ARDS from classical ARDS and influenza-related lung injury [6,66]. These findings help explain observed differences in vascular pathology, microthrombosis, and treatment responses and provide a rationale for targeted immunomodulatory and endothelial-protective therapies in selected patient populations.

Together, these recent advances reinforce the central themes of this review: viral ARDS is biologically heterogeneous, pathogenesis varies across viral etiologies, and future therapeutic progress will likely depend on integrating mechanistic insights with phenotype-guided clinical management rather than relying on uniform treatment strategies.

## Conclusions

ARDS related to emerging and pandemic respiratory viruses, including SARS-CoV-2 and highly pathogenic influenza A strains, remains a major clinical challenge with substantial mortality. Virus-induced ARDS reflects marked biological heterogeneity driven by the interplay of direct viral injury and dysregulated host responses, often involving hyperinflammation and cytokine-mediated tissue damage. Improving outcomes, therefore, depends on continued clarification of the mechanisms linking viral replication, epithelial-endothelial barrier disruption, inflammation, thrombosis, and downstream fibrosis.

Management must remain multifaceted and etiology aware. Supportive care with lung-protective ventilation is foundational, but optimal treatment increasingly requires appropriate timing and selection of antiviral therapies together with immunomodulatory or endothelial-targeted strategies, recognizing that benefits and harms vary across viral etiologies and patient subgroups. Future gains will likely come from precision approaches that identify distinct ARDS phenotypes early and evaluate targeted interventions designed to limit viral burden while modulating immune and immunothrombotic dysregulation, with the goal of reducing both acute mortality and long-term disability.
